# Guided Internet-Delivered Treatment for Depression: Scoping Review

**DOI:** 10.2196/37342

**Published:** 2022-10-04

**Authors:** Line Børtveit, Anders Dechsling, Stefan Sütterlin, Tine Nordgreen, Anders Nordahl-Hansen

**Affiliations:** 1 Faculty of Health, Welfare and Organisation Østfold University College Halden Norway; 2 Faculty of Health Sciences Department of Behavioral Sciences Oslo Metropolitan University Oslo Norway; 3 Department of Education, ICT, and Learning Østfold University College Halden Norway; 4 Faculty of Computer Science Albstadt-Sigmaringen University Sigmaringen Germany; 5 Division of Psychiatry Haukeland University Hospital Bergen Norway; 6 Departement of Global Public Health and Primary Care University of Bergen Bergen Norway

**Keywords:** web-based therapy, computer-assisted therapy, internet, digital interventions, major depression, mental health, mobile phone

## Abstract

**Background:**

Studies on guided internet-delivered treatment have demonstrated promising results for patients with depressive disorder.

**Objective:**

The aim of this study was to provide an overview of this research area and identify potential gaps in the research.

**Methods:**

In this scoping review, web-based databases were used to identify research papers published between 2010 and 2022 where guided internet-delivered treatment was administered to participants with depressive disorders, a standardized rating scale of depressive symptoms was used as the primary outcome measure, and the treatment was compared with a control condition.

**Results:**

A total of 111 studies were included, and an overview of the studies was provided. Several gaps in the research were identified regarding the design of the studies, treatments delivered, participant representation, and treatment completion.

**Conclusions:**

This review provides a comprehensive overview of the research area, and several research gaps were identified. The use of other designs and active control conditions is recommended. Future studies should provide access to treatment manuals, and more replications should be conducted. Researchers should aim to include underrepresented populations and provide reports of comorbidities. Definitions of adequate dosage, reports of completion rates, and reasons for treatment dropout are recommended for future studies.

## Introduction

### Depressive Disorder and Treatment Over the Internet

The number of people with access to the internet is considerable and increasing also in low-income countries [1]. As the number of people with access to the internet is sizable, internet-delivered treatment has the potential to reach more patients, be easily accessible, and be less time-consuming for the therapist per patient than more resource-intensive face-to-face therapies [2-4]. The COVID-19 pandemic has also demonstrated that situations might arise when face-to-face therapy can be less available, thus suggesting the benefits of internet-delivered treatments [5].

Depressive disorders, or clinical depression, are associated with great personal distress and high societal costs [6]. Common features of depressive disorders according to the Diagnostic and Statistical Manual of Mental Disorders [7] are sad, empty, or irritable mood and somatic and cognitive changes that decrease the ability to function at work and at home. As the number of people with clinical depression is increasing, there is a demand for effective and accessible treatments [8]. Meta-studies on internet-delivered psychological treatment show promising results for adults with mild or moderate depressive disorder [9-12], and this treatment option could be an accessible and less resource-intensive alternative to traditional face-to-face treatment [10].

Internet-delivered treatments typically involve working with different modules composed of reading assignments and tasks in a self-help format [13]. Treatments are often based on evidence-based treatment for depressive disorder; *cognitive behavioral therapy* (CBT); or other psychological treatments such as *acceptance and commitment therapy*, *problem-solving therapy*, positive psychology, or psychodynamic therapy [10,11,14]. These treatment options are accessible digitally through web-based programs such as web pages and phone apps and are available to the patient wherever there is internet access.

In *therapist-guided internet-delivered treatment*, additional guidance and support are provided by therapists, coaches, or other professionals over the internet via chats, emails, and telephone [14-16]. In unguided treatment programs, the patients usually have access to the treatment content, and some programs provide automated prewritten feedback. In guided treatment programs, a therapist often plays an active role in the treatment and guides the patient (or research participant) through the program. Studies have shown that both treatment formats could be effective and that a guided program is not necessarily superior to an unguided program [17,18] but that the inclusion of guidance could have an impact on treatment adherence [19].

A few recent meta-studies have focused primarily on guided treatments. Karyotaki et al [11] found guided internet-delivered treatments to have positive treatment effects, especially for patients with more severe depressive symptoms at baseline. Chan et al [12] found effectiveness in reducing depression and anxiety and lower attrition rates than previously reported for guided internet-delivered CBT. Carlbring et al [20] compared guided internet-delivered CBT with face-to-face therapy and concluded that the 2 treatments were equally effective for depression and several other psychiatric and somatic disorders.

To our knowledge, no recent review aimed at providing an overview and identifying potential research gaps focusing on guided treatments for patients with depressive disorder has been conducted.

### Scoping Review

A scoping review is a method used to provide an overview of a research area. The *PRISMA-ScR (Preferred Reporting Items for Systematic Reviews and Meta-Analyses extension for Scoping Reviews*) provides a framework with guidelines for conducting and reporting scoping reviews [21,22]. A key capacity of a scoping review is that it can synthesize research and describe what evidence is known and available in a confined area of research in the form of publicized empirical accounts. However, another important aspect of a scoping review is that it can inform and contribute to a research field by identifying potential gaps in knowledge.

In this scoping review, we focused on 4 overarching aspects of the included studies. We focused on (1) the design (and methods), (2) the treatments delivered, (3) the characteristics of the participants, and (4) how treatment completion was reported in the studies. We then considered potential gaps in the literature that should be taken into account in future research on internet-delivered treatment for depression.

### Type of Design

In previously published reviews and meta-analyses on internet-delivered treatment for depression, *randomized controlled trials* (RCTs) have been the most prevalent methodological design [11,14-16]. Etzelmueller et al [10] reviewed guided internet-delivered CBT focusing only on nonrandomized pre-post designs to evaluate the intervention effectiveness in a clinical setting and found evidence of both acceptability and effectiveness. Effectiveness studies investigating potential effects of treatment programs in a *real world* clinical setting could provide stronger conclusions about expected treatment outcomes than efficacy studies conducted in a controlled environment with more “ideal settings.” In their systematic review of the implementation of internet-delivered treatments for depression in public health primary care settings, Rodriguez-Pulido et al [23] concluded that there were few studies outside the United Kingdom where this was investigated. Given the high prevalence of depression and the need for clinical evidence on the effectiveness of known treatment programs, it is of high relevance that a review of the available research and an evaluation of the study setting are warranted. Other factors regarding how studies are designed are also recommended for inclusion in a scoping review. These factors include descriptions of the comparator or control conditions (if used) in the intervention, the aims of the study, and the outcome measures used [24]. Including a variety of designs in a scoping review has the benefit of providing a more comprehensive overview of the research area [25]. At the same time, it is recommended to limit the inclusion of studies using designs related to answering the research questions (RQs) in the review [24]. In some research areas, the number of studies published can be quite extensive. Hence, some type of exclusion criterion is needed. This scoping review focused on efficacy or effectiveness studies that presuppose experimental and quasi-experimental designs, and therefore, studies with at least one control condition were included.

### Type of Treatment

Reports on the properties of the treatment, duration, and other methodological choices are recommended for inclusion in scoping reviews [25]. These factors could provide a useful overview of the treatments delivered and contribute to the identification of potential research gaps. Internet-delivered treatments based on CBT have been the principal subject matter of several newer reviews [10,12,20,26,27] and have even been the most prevalent treatment approach in reviews where other treatment approaches were also considered [11,14,16]. For example, Karyotaki et al [11], who focused exclusively on guided internet-delivered treatment for depression, showed that most treatments tested were based on CBT (>70%) or problem-solving therapy. Tokgöz et al [16] and Garrido et al [14] reported similar findings as well.

When reviewing treatments, easy accessibility to treatment manuals is beneficial for transparency purposes and for making comparisons between studies feasible [28]. In addition, access to manuals is beneficial for future replication studies as well as for developing new treatments in which content from previously tested treatment programs is used. A review of trials in which psychological treatments were delivered for common mental disorders showed lower rates of access to the treatment manuals [28], and similar results were found in a review of internet-delivered CBT for adults in treatment for depression and anxiety [10]. There are, to our knowledge, no recently published reviews where the accessibility of treatment manuals in studies on guided internet-delivered treatment for depression is investigated.

### Participant Characteristics

In highly tailored treatments with relevant content directed at patients with depression, detailed descriptions of the participants are important. Many recently published reviews and meta-analyses on internet-delivered treatment have focused on treatments directed at adults with depression [10,11,16,20], and several have focused on 1 particular population group (eg, perinatal women with anxiety and depression [15], young people with anxiety or depression or a combination of both [14], participants aged >50 years [29], or participants living in lower-income countries [30]). Given the focus on specific populations, these reviews provide limited insight into the area of guided internet-delivered treatment for depression as a whole. In this review, we wanted to provide an overview of sex, age, and location (country) representation in the studies to investigate whether some populations are over- or underrepresented.

Participants from Western countries have been overrepresented in previous reviews [10-12,14,15,20,26], with few or none of the included studies recruiting participants from countries outside the Western world. Martínez et al [30] reviewed studies with participants from lower-income countries and found only 6 studies.

There is a high prevalence of comorbid disorders to depression [31] as well as of comorbid anxiety disorder [32-34]. If the goal is to create effective treatments for the general population, it would also be advantageous to report whether the treatments were tested on participants with other diagnoses or additional comorbidities.

A synthesis including all studies regardless of the participant group in focus could contribute to the identification of potential research gaps in the represented populations.

### Reports of Treatment Completion

Nonadherence and dropout rates have been reported to range from 0% to 75% in digital CBT treatments for depression [35], and the challenges of nonadherence have been discussed in several meta-studies [36-39]. Discontinuation of treatment hinders its effective delivery. First, patients not receiving an “adequate dose” of the therapy might not improve or may even experience a worsening of symptoms. Furthermore, limited resources (eg, therapy appointments) go to waste if patients discontinue treatment before an adequate dose is delivered, which could affect the ability of others to receive access to needed services and treatment [40,41]. With internet-delivered treatments, adherence has been defined as the extent to which individuals experience or engage with the content of the treatment [36,42]. Measures of adherence have included reports on the number of log-ins to the program, modules completed, time spent on the web, completion of different activities or use of web-based tools, posts made, pages viewed, replies to emails, forum visits, self-reports of completion of offline activities, and print requests made [43]. The adherence rate is commonly defined as the percentage of participants who complete treatment [39], which could include those participants who complete either the entire treatment or a specified amount of the lessons, modules, or exposure [44].

Completion of treatment as a measure of treatment adherence has been discussed as a problematic measure as treatment drop out might be the result of improvement [45], and “exposure” to a higher dosage of treatment does not necessarily mean better results [46]. In addition, participants might also use the technology in other ways than the developers intended [46], which can thus further skew the measured outcomes and interpretation of the results. The reasons or motivation for choosing the internet-delivered treatment could be of more importance for the results than the frequency or duration of exposure [46]. The adequate dose necessary to experience improvement could also vary among the different participants and user groups [45].

There is a lack of consensus on how to report treatment adherence [43,44]. Given that completion of treatment is described as a commonly used measure of adherence [39], this was also a focus of this scoping review. Reasons given (if reported) by participants for treatment discontinuation were also charted, which has often been described as not reported in many studies [39,44]. Understanding treatment discontinuation can provide useful information for treatment development and patient care and guide the design of future studies.

### Aim and RQs

The aims of this scoping review were to analyze and describe currently available quantitative research on guided internet-delivered treatment for participants with depressive disorder, provide insight into this research area, and identify potential gaps in research.

We posed the following four RQs: (1) How are the studies designed? (RQ 1), (2) What types of treatments are delivered? (RQ 2), (3) What are the characteristics of the participants in the studies? (RQ 3), and (4) What is the treatment completion in the various studies? (RQ 4).

## Methods

### Scoping Review

The PRISMA (Preferred Reporting Items for Systematic Reviews and Meta-Analyses) guidelines [21] for conducting systematic scoping reviews, described by Peters et al [24], were followed. Given that the aims of this scoping review were to describe the research and identify the potential research gaps, no quality assessment of the included studies was conducted.

### Eligibility Criteria

Quantitative studies published in peer-reviewed journals between January 1, 2010, and June 4, 2022, were eligible for inclusion. Full-text accessible publications in the English or Scandinavian languages were included.

Even though this scoping review could have included noncontrolled studies, we restricted the inclusion criteria to controlled experimental studies where at least one condition stipulated that participants received guided internet-delivered treatment to reduce the potential extensiveness of this review. Reviews, meta-analyses, conceptual articles, and nonempirical articles were excluded.

A standardized rating scale for depressive symptoms (eg, Center for Epidemiological Studies-Depression [48], Patient Health Questionnaire-9 [49], and Montgomery-Åsberg Depression Rating Scale [50]) had to be used as the primary outcome measure throughout the study. To secure a comparable group of participants and limit the focus of the review, only studies in which the participants initially scored mild or moderate for depression were eligible for inclusion. Studies focused on preventing depressive disorder or promoting psychological well-being were excluded. Studies including participants with comorbid disorders were accepted as long as depressive symptoms were a primary outcome measure. We implemented no limitations regarding participant age, which typically has been the case in previous reviews [10,11,16,20,51]. We included studies with treatments directed at children as well as teenagers and young adults, thus allowing for comparisons between age groups, providing insight into age representation in the studies, and identifying potential studies that were previously excluded. We also assumed that younger people were more adept at using digital solutions, and therefore, there would be a vast number of studies with treatments directed at this subpopulation.

For this scoping review, treatments had to be delivered over the internet and accessible wherever the participants had access to the internet (the device used by the participants was not decisive, and both phone apps and web page–available interventions were included). Studies in which the participants were required to be in a specific location to have access to the treatment (eg, at home, in school, or at the psychologist’s office) were excluded. The guided internet treatments had to be a stand-alone treatment and not a supplement to face-to-face treatments. Only treatments in which the participants received guidance from a *trained* guide during the treatment were eligible. To be *trained* was defined as having a degree in psychology, social work, or nursing or other relevant education or being a student, in training, or a layperson and receiving special courses or training in the methods and supervision from a trained professional. The guidance had to be implemented during the entire treatment and not limited to measures and testing or reminders to complete web-based tasks.

See Multimedia Appendix 1 for the coding manual used.

### Information Sources

The second author (AD) conducted a systematic search on June 4, 2022. The scientific databases PubMed, Scopus, PsycINFO, MEDLINE, and ERIC were searched. See Multimedia Appendix 2 for details about the search.

A total of 28 published systematic reviews on internet-delivered treatment (Multimedia Appendix 3 [10,11,14-16,20,27,29,30,51-69]) were hand searched by LB for relevant studies not identified in the literature search. The identified studies were screened using the same criteria applied when screening the database records.

### Selection of Sources of Evidence

After removal of duplicates using the EndNote (version 20; Clarivate Analytics) duplicate finder followed by manual removal, the results were imported into the systematic review research tool Rayyan (Rayyan Systems Inc) [70] and screened by the first author (LB) for inclusion. Screening was performed by first reading the title and abstract and thereafter by screening the full-text articles. For studies where the eligibility was unclear, the fifth author (ANH) was consulted, and an agreement was reached after discussion between the 2 authors.

### Data Items

#### Overview

The data were extracted and charted by LB. Author names, title of the article, journal, and year of publication were extracted as well as the items described in the following sections related to the 4 RQs. A detailed description of the extraction and charting of the data is provided in Multimedia Appendix 4 [7,71,72].

#### RQ 1: How Are the Studies Designed?

To answer this question, information about research design, control conditions, whether the studies were considered to be efficacy or effectiveness studies, and the outcome measures collected in the studies was charted.

#### RQ 2: What Types of Treatments Are Delivered?

To answer this question, information about the treatments, including treatment approaches, accessibility of treatment manuals, names of the treatments, by whom the guidance was delivered, and the duration of the treatment, was charted.

#### RQ 3: What Are the Characteristics of the Participants in the Studies?

To answer this question, information about the participants was charted. This included the number of participants, sex, mean age, age group in focus, reports of comorbid disorders, and the countries from which the participants were recruited.

#### RQ 4: What Is the Treatment Completion in the Various Studies?

To answer this question, information about treatment completion was charted, including if a definition of treatment completion was provided, the number of participants defined as completers, and reasons for discontinuation of treatment (if provided).

## Results

### Selection of Sources of Evidence

**Figure 1:**
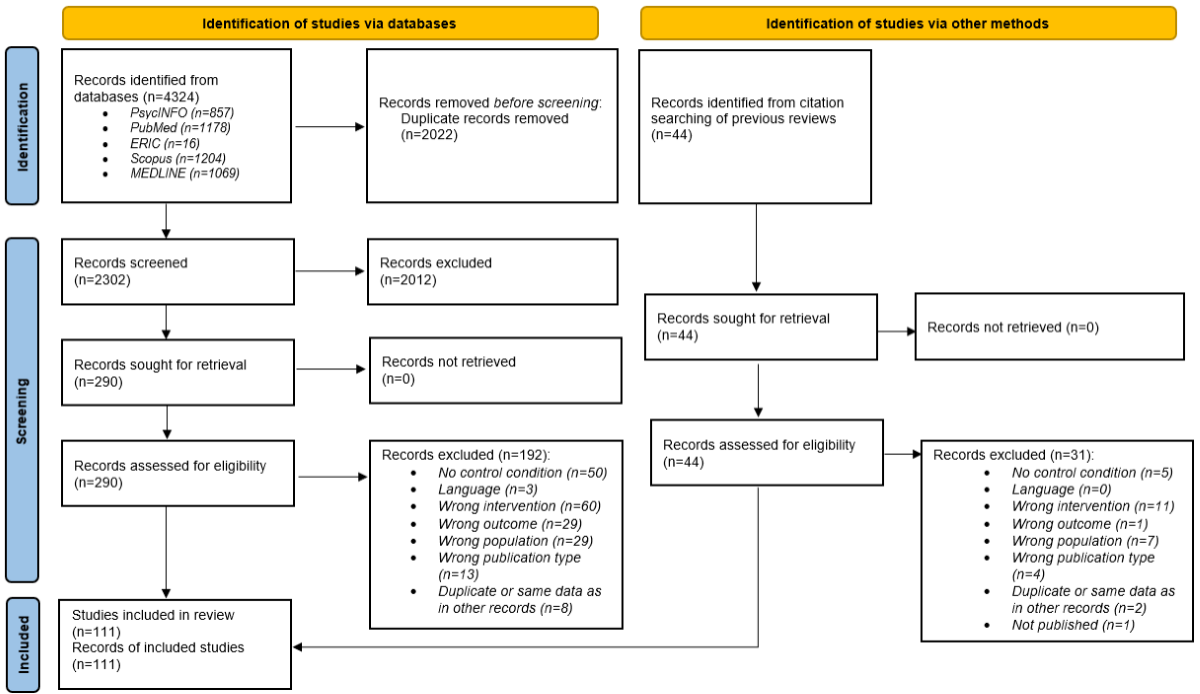
Results from the screening process and literature search. Flow diagram showing inclusion and exclusion strategy.

The results of the screening process are presented in Figure 1. The literature search resulted in 4324 records, of which 98 (2.27%) were included in this scoping review.

The hand search of 28 published reviews yielded 13 additional studies. Thus, the total number of studies included in this review was 111.

### Synthesis of Results

In Multimedia Appendix 5 [17,73-182], the data charted from and the characteristics of the different sources of evidence are presented. Multimedia Appendix 5 provides an overview of the 111 included studies.

#### Study Design and Methods

The first RQ—“How are the studies designed?”—was related to what types of study designs and methods that were applied in the studies.

##### Study Design

Most publications (110/111, 99.1%) were RCTs. A total of 0.9% (1/111) were prospective cohort studies.

##### Control Condition

A total of 52.3% (58/111) of the studies included at least one active control condition (eg, access to other treatments or variations of the treatment). Of the 58 studies considered, 38 (66%) solely had active controls, whereas the other 20 (34%) used a combination of active and inactive controls. In addition, 47.7% (53/111) of the included studies had exclusively inactive control conditions (eg, no treatment, waitlist, attention control, or treatment as usual).

##### Efficacy and Effectiveness

A total of 81.1% (90/111) of the studies were defined as effectiveness (43/111, 38.7%) or efficacy studies (47/111, 42.3%). The remaining 18.9% (21/111) did not provide a definition.

##### Outcome Measure

A total of 14 different psychometric scales were used to measure depressive symptoms in the studies. In 17.1% (19/111) of the studies, a combination of different measurement scales was used as a primary outcome measure. In Multimedia Appendix 5, all relevant measurement scales used in the studies are charted. The 3 most applied outcome measurement scales for depressive symptoms were the Patient Health Questionnaire-9 [49], Beck’s Depression Inventory [183], and the Center for Epidemiological Studies-Depression scale [48].

##### Research Gaps in Study Design and Methods

Table 1 provides an overview of the identified research gaps with regard to how the studies were designed.

**Table 1:** Identified gaps in the reviewed literature regarding design.

Identified gap	Reason	Report	Percentage of studies reporting
Lack of diversity in research design	Few studies with designs other than RCT^a^	Only 1 study with other design (<1%)	100% of the studies reported the design used
Need for more studies with an active control condition	Relatively many studies with an inactive control condition	47.7% of the studies had only an inactive control condition	100% of the studies described the control condition
Large variations in the outcome measure used	Several different measurement scales and combinations of these were used	14 different measurement scales were used; 19 studies where combinations of different measurement scales were used	100% of the studies reported the outcome measure used

^a^RCT: randomized controlled trial.

#### Treatment Characteristics

The second RQ—“What types of treatments are delivered?”—focused on what types of treatments were delivered and how the treatments were designed.

##### Treatment Approaches

Table 2 provides an overview of all the approaches used. A total of 123 treatments were tested. In 6.3% (7/111) of the studies, treatments were based on a combination of different approaches (eg, behavioral activation and physical activity [73] or problem-solving therapy and cognitive therapy [74]). A total of 1.8% (2/111) of the studies included several experimental arms that received treatments based on different approaches. In Kladnitski et al [75], one group received CBT, one group received CBT in combination with mindfulness, and one group received mindfulness exclusively. In the study by Stiles-Shields et al [76], one group received behavioral activation, and one group received CBT. For an overview of the treatment approaches used in the different studies, please see Multimedia Appendix 5.

**Table 2:** Treatments tested as stand-alone approaches, in combination, or as one of several arms in the studies (n=123).

Treatment approaches	Tests, n (%)
Acceptance and commitment therapy	4 (3.3)
Behavioral activation	9 (7.3)
Cognitive behavioral therapy	82 (66.7)
Information	1 (0.8)
IPDT^a^: affect-focused psychodynamic psychotherapy	2 (1.6)
Life review therapy	1 (0.8)
Mindfulness	4 (3.3)
Physical activity	3 (2.4)
Problem-solving therapy	13 (10.6)
Psychodynamic treatment	1 (0.8)
Sleep diary	1 (0.8)
Social cognitive theory	1 (0.8)
Stress process model	1 (0.8)

^a^IPDT: internet-based psychodynamic therapy.

##### Treatment Manual

None of the studies provided easy access to the entire treatment manual. Many studies included brief descriptions of the treatment, such as a table describing the treatment content. Several studies referred to previous publications for a more detailed description of the treatment, but even in those cases, the manuals were not accessible. In all, 2.7% (3/111) of the studies provided more extensive treatment descriptions in the study protocol [77] or in the supplementary material or appendix [78,79].

##### Name of Treatment

In all, 71.2% (79/111) of the studies referenced a named treatment program. The most frequently identified treatment programs were different versions of The Wellbeing Course (8/111, 7.2%) [80] and The Sadness Program (7/111, 6.3%; including culturally adapted versions) [184].

##### Guidance

Only 0.9% (1/111) of the studies [81] did not report the background or training of the guides. Trained professionals delivered the guidance in 67.6% (75/111) of the studies. Most of the guidance was delivered by psychologists, social workers, and nurses, but there were also studies where guidance support was delivered by lay counselors [82] and nonprofessional volunteering telephone counselors [83] who did receive some training beforehand. A total of 6.3% (7/111) of the studies had research staff and authors providing the guidance, and 25.2% (28/111) of the studies had students or professionals in training supporting the participants.

##### Duration

Most studies (101/111, 91%) described the duration of the treatment in weeks, months, or years, and often the participants had to complete 1 module or session per week. A total of 3.6% (4/111) of the studies described the number of sessions or modules that should be completed without reference to time [84-87]. In total, 5.4% (6/111) of the studies did not describe the duration of the treatment [88-93]. The longest treatment lasted for 52 weeks [78], and the shortest lasted for 3 weeks [94]. For the 91% (101/111) of the studies where it was possible to chart duration in weeks, the mean treatment duration was 9.3 (SD 5.6) weeks with the range being 3 to 52 weeks, and the median was 8 weeks.

##### Research Gaps Regarding the Treatments Delivered

In total, 2 research gaps regarding types of treatments delivered were identified in the included studies. First, none of the studies provided easy access to a treatment manual.

Second, there was large variation in the treatment programs used. Only 47.7% (53/111) of the studies tested treatments that were used in at least one other study included in this review. This points to a lack of replications. However, only 71.2% (79/111) of the included studies reported the name of the treatment program, and unidentified replication studies are possible.

#### Participant Characteristics

The third RQ—“What are the characteristics of the participants in the studies?”—was related to the participants in the included studies.

##### Number of Participants

There were a total of 11,851 participants across all studies. Not all participants from all studies were included as some study arms did not fulfill the inclusion criteria (eg, studies where a comparison group did not receive web-based treatment or had a diagnosis other than depression). The mean number of participants with relevant conditions was 106.8 (SD 151.83, median 52, range 9-949).

##### Sex of Participants

Table 3 summarizes the representation of sex in the studies. The studies had an average of 74% female participants (median 76.6%, range 12.4%-100%). In 7.2% (8/111) of the studies, sex was not reported [76,95,96] or was reported for the total sample but not separated for the different conditions [97-101]. In total, 8.1% (9/111) of the studies included only women and had treatments directed at maternal depression [84,87,88,102-105], patients with breast cancer [106], or female adolescents [107].

A total of 7.2% (8/111) of the studies included <50% women. Of these 8 studies, 6 (75%) had participants with comorbid disorders (heart problems [108-111], HIV [112], and kidney diseases [113]), 1 (12%) was directed at a Kurdish population in Sweden [114], and 1 (12%) was directed specifically at army Veterans [115].

**Table 3:** Female participants (N=111).

Percentage of female participants	Publications, n (%)
0% to 20%	2 (1.8)
21% to 40%	4 (3.6)
41% to 60%	7 (6.3)
61% to 80%	60 (54.1)
81% to 100%	35 (31.5)
NR^a^	3 (2.7)

^a^NR: not reported.

##### Age of Participants

A total of 5.4% (6/111) of the studies did not report the participants’ ages [76,85,88,101,104,116]. The mean age across the remaining 94.6% (105/111) of the studies was 40.9 (SD 11.6, median 42.3, range 15.8-69.6) years.

##### Age Group in Focus

None of the included studies had treatments directed at children. A total of 9.9% (11/111) of the studies included teenagers or young adults. Most studies (96/111, 86.5%) had treatments directed at adults. A total of 3.6% (4/111) of the studies included solely older adults [117-120].

##### Comorbid Disorders

In 24.3% (27/111) of the studies, comorbidities or secondary diagnoses besides depressive disorder were not reported. In 26% (7/27) of these studies, comorbidities were stated as being included, but no description of these comorbidities was provided. Most studies (68/111, 61.3%) included participants with other Diagnostic and Statistical Manual of Mental Disorders, Fourth Edition (DSM-IV), Axis I disorders [7] (eg, anxiety disorders, posttraumatic stress disorder, and eating disorders) in combination with depression.

In all, 19.8% (22/111) of the studies included participants diagnosed with both depression and a physical disease (eg, renal diseases [78,113], breast cancer [106], and multiple sclerosis [121]). In 5.4% (6/111) of the studies, participants were diagnosed with a combination of physical diseases and mental disorders or other DSM-IV Axis I disorders in addition to depression (eg, cancer, anxiety and stress [95,122], or diabetes and anxiety [123].

##### Countries of Recruitment

See Table 4 for an overview of all the countries in which the participants were recruited. Most studies (104/111, 93.7%) were conducted in and included participants from Western countries, but non-Western countries such as Iran [107], Colombia [126], and Indonesia [82] were also represented. In total, 2.7% (3/111) of the studies included a population that was considered to have a culturally different background than most participants recruited. These culturally different populations were Turkish migrants in the Netherlands [124], the Kurdish population in Sweden [114], and Chinese Australians [127].

**Table 4:** Countries where participants were recruited (N=111).

Countries	Publications, n (%)
Australia	20 (18)
Brazil and Peru	1 (0.9)
Canada	6 (5.4)
China	1 (0.9)
Colombia	1 (0.9)
Finland	2 (1.8)
Germany	7 (6.3)
Indonesia	1 (0.9)
Iran	1 (0.9)
Ireland	1 (0.9)
Netherlands	15 (13.5)
Norway	1 (0.9)
Romania	1 (0.9)
Spain	2 (1.8)
South Korea	1 (0.9)
Sweden	26 (23.4)
Switzerland	2 (1.8)
Switzerland and Germany	1 (0.9)
United Kingdom	6 (5.4)
United States	15 (13.5)

##### Research Gaps Regarding the Participants in the Studies

In Table 5, research gaps with regard to the participants in the studies are identified.

**Table 5:** Identified gaps in the reviewed literature regarding characteristics of the participants.

Identified gap	Reason	Report	Percentage of studies reporting
Female participants overrepresented	More female than male participants in the studies	The studies had an average of 74.1% female participants	Sex was reported in 97.3% of the studies
Lack of studies directed at teenagers and young adults	Few studies of treatments directed at teenagers or young adults	11 studies directed at teenagers or young adults	100% of the studies reported or provided sufficient information to draw conclusions about age group in focus
Lack of studies directed at older adults	Few studies of treatments directed at older adults	4 studies included solely older adults	100% of the studies reported or provided sufficient information to draw conclusions about age group in focus
Lack of reports of comorbid disorders	Several studies where comorbid disorders were not described in detail or reported	24.3% of the studies did not include reports of comorbid disorders	75.7% of the studies reported comorbidity
Non-Western participants underrepresented	Few studies with participants from non-Western countries	6.3% of the studies recruited participants from non-Western countries	100% of the studies reported or provided sufficient information for conclusions about origin

#### Treatment Completion

The fourth RQ—“What is the treatment completion in the various studies?”—was related to treatment completion in the studies, including if the researchers provided a definition of adequate dosage for the treatment to be considered completed, the number of participants completing the treatment, and reasons provided for treatment dropout.

##### Definitions of Treatment Completion

Of the 111 studies, 22 (19.8%) provided a clearly stated definition of treatment completion.

Completion was often defined as completing all modules or sessions [77,87,128] or a majority of the modules (eg, 5 of 8 modules [82]). A total of 0.9% (1/111) of the studies defined completion as having started in the last module [129].

##### Number of Completers

Overall, 80.2% (89/111) of the studies provided information about the number of participants who completed all the treatment modules or lessons or fulfilled a stated definition of treatment completion. See Table 6 for an overview of the percentage treatment completers in the 80.2% (89/111) of studies. In 34% (30/89) of these studies, <50% of the participants completed the treatment. In 66% (59/89) of these studies, >51% of the participants completed the treatment; of these 59 studies, there were 5 (8%) with >90% of the participants completing the treatment [118,130-133].

**Table 6:** Treatment completers (N=89).

Percentage of treatment completers	Publications, n (%)
0% to 20%	10 (11)
21% to 40%	14 (16)
41% to 60%	21 (24)
61% to 80%	26 (29)
81% to 100%	18 (20)

##### Reasons for Noncompletion

A total of 15.3% (17/111) of the studies clearly stated reasons why participants did not complete the treatment. In 100% (17/17) of these studies, several reasons for noncompletion were reported. The most reported reason was that some of the patients lacked time to complete the treatment, as reported in 71% (12/17) of these studies. Treatment not meeting personal needs (10/111, 9%) and personal problems or sickness (10/111, 9%) were also described by patients as reasons for not completing. In 8.1% (9/111) of the studies, some of the participants dropped out without providing reasons for noncompletion. See Table 7 for details from the 15.3% (17/111) of the studies.

In the remaining studies (94/111, 84.7%), reasons for noncompletion were either not stated (88/94, 94%) or not clearly stated (6/94, 6%). During coding, we noted that several studies provided reasons for attrition (dropout from the study; eg, failure to complete follow-up questionnaires), but these data were not charted in this review.

**Table 7:** Reasons participants provided for noncompletion of the treatment in the 17 studies where this information was described.

Reason	Study																
	Boele et al [139], 2018	Boeschoten et al [121], 2017	Geraedts et al [74], 2014	Heller et al [87], 2020	Høifødt et al [138], 2013	Karyotaki et al [144], 2022	Kenter et al [137], 2016	Kleiboer et al [136], 2015	Lappalainen et al [132], 2015	Nadort et al [113], 2022	O’mahen et al [88], 2014	Pots et al [145], 2016	Preschl et al [134], 2011	Ström et al [81], 2013	Van der Zweerde et al [98], 2019	Wagner et al [135], 2014	Westerhof et al [79], 2019
No reason provided	✓^a^		✓	✓	✓		✓	✓						✓	✓	✓	
Not meeting personal needs	✓	✓		✓	✓	✓	✓	✓		✓				✓		✓	
Recovery	✓			✓	✓		✓	✓			✓		✓			✓	
Preferred other treatment or part of the treatment	✓	✓		✓	✓			✓								✓	
Technical difficulties	✓	✓				✓	✓	✓								✓	
Lack of time	✓	✓	✓	✓	✓	✓		✓			✓	✓	✓	✓	✓		
Motivation				✓		✓	✓	✓		✓	✓		✓			✓	✓
Personal problems or sickness	✓	✓			✓		✓		✓	✓		✓		✓	✓		✓

^a^✓: indicates that at least one participant in the study stated the reason as a cause for treatment noncompletion.

##### Research Gaps Regarding Completion of Treatment

In Table 8, research gaps regarding the definition of treatment completion, the rate of completion, and the reasons provided for treatment dropout are presented.

**Table 8:** Identified gaps in the reviewed literature regarding treatment completion.

Identified gap	Reason	Report	Percentage of studies reporting
Lack of clear definitions of treatment completion	Few studies where completion was defined	A minority (n=22) of the studies provided a clearly stated definition of adequate or necessary dosage of the treatment for it to be considered completed	19.8% of the studies provided definitions of treatment completion or adequate dosage
Lack of reports of treatment completion	Several studies without reports of the number of participants who completed the treatment	89 studies provided information about the number of completers (completed all the treatment modules or fulfilled a stated definition)	80.2% of the studies reported the number of participants who completed the treatment
Lack of reports on reasons for treatment dropout	Most studies did not provide reasons for participants dropping out of treatment	17 studies clearly stated reasons for noncompletion of the treatment	15.3% of the studies reported reasons for discontinuation of treatment

## Discussion

### Summary of Evidence

In this scoping review, we identified 111 studies published between 2010 and 2022 on guided internet-delivered treatment for participants diagnosed with depression.

#### Study Design and Methods

There was a high degree of uniformity regarding research designs, with 99.1% (110/111) of the studies using RCTs; these results are similar to results from previously published reviews on internet-delivered treatment for depression [11,14-16]. We anticipated to identify controlled experimental studies without randomization (eg, comparing the effects for men vs women), including studies with multiple single-case designs with within-person control or other variations of research designs. Even though we did not exclude nonrandomized studies, our findings might still be a result of the quite strict inclusion criteria, such as the studies needing to include at least one control condition. A scoping review including noncontrolled studies might produce results in which further information about the treatments is identified [47] and could be useful for updating treatments in an applied setting [10].

There were an equal number of efficacy and effectiveness studies in the part of our sample where this was reported. Given that one of the inclusion criteria stipulated that participants should have depressive disorder, one might expect the identification of a higher number of effectiveness studies conducted with patients in clinical settings. However, the criteria that the studies had to have at least one control condition may have indirectly led to the exclusion of these types of studies. In a clinical setting, it might have been viewed as impractical or even unethical to not offer the same conditions to all patients. This might also explain why a large amount of the studies had an active control condition or a combination of active and inactive control conditions.

Even though most studies (58/111, 52.3%) had at least one active control condition, 47.7% (53/111) had an inactive control condition. As active control is preferable [185], this represents a gap in the research, and future studies should aim to be designed with an active control condition.

There was quite a large variation regarding outcome measures considering that only standardized measurement scales were accepted. Many studies used combinations of various scales. A possible reason might be that studies were conducted by a variety of research teams in different countries and that personal preferences and traditions could influence such decisions. The use of different outcome measures might not be problematic, and treatment effects could still be compared, but it could represent a possible scaling issue if there are large variations in how depression is defined. There is a risk that different phenomena were measured in the different studies, where some behaviors defined as depressive symptoms in one study might not be considered so in another study. In addition, what is used as primary outcome measures varies and in many instances, is not specifically mentioned or defined as the primary outcome in the trial.

#### Treatment Characteristics

We identified several different treatment approaches, but most studies (81/111, 73%) used some variation of CBT. This might represent the standard treatments recommended for patients with depression [186] and is also similar to the findings of a previous review on guided internet-delivered treatment for depression [11]. This could be an indicator that the treatments were implemented based on evidence-based studies and results.

With no publications where the treatment manual was easily accessible, the results are weaker than in previous reviews [10,28], and this gap in research should be addressed in future studies. The lack of accessibility to treatment manuals makes the studies less transparent as well as making comparisons and replications more difficult.

A large variety of treatment programs was used, which could indicate that more studies using the same programs would be beneficial as opposed to every research team investigating a new program. However, lack of accessible treatment manuals and with several unnamed treatments used in the included studies, it is possible that some studies used the same treatment programs without it being identified in this review.

Trained professionals accounted for most of the guidance, which was expected for studies with participants diagnosed with clinical depression, and these findings were in line with previously published reviews [10,11]. However, research staff and students were also used as guides in several studies (35/111, 31.5%).

In line with the common duration of face-to-face sessions in CBT for depression [187,188], most treatments in our sample lasted between 5 and 12 weeks.

#### Participant Characteristics

There were large variations in the number of participants included in the studies. This scoping review evaluated studies with a combined total of >11,000 participants and thus provided a large demographic profile of the participants studied.

Female participants were overrepresented in the studies, with an average of 74%. Although this might indicate a sex prevalence of depression, treatments should be tested for all potential target populations. Those studies directed at participants diagnosed with both depression and a comorbidity or physical disease (eg, heart failure [108]) had more male participants. Our review also identified 8.1% (9/111) of the studies directed at women only, mostly with the aim of reducing maternal depression. Studies on treatments directed at maternal depression have often been excluded from previous reviews [52] or reviewed separately [15].

Participants from non-Western countries were also underrepresented in the studies. Given the increasing access to internet-connected devices and the steady rise in the number of people in need of treatment in these countries [189], more studies with non-Western participants would be advantageous.

Studies with treatments specifically directed at teenagers or young adults and older adults were underrepresented in the research. However, there was some overlap in some of the included studies in which several studies recruited and enrolled participants aged <30 years and >60 years. The prevalence of depressive disorders is higher in young adults than in any other age group in high-income countries [189], and providing and testing treatments for this targeted group should be prioritized.

We identified several studies with computerized treatments directed at children. However, treatments directed at children often involved working together with a therapist or coach at school or in a therapist’s office, and these studies were excluded from our review because of the limited accessibility and requirement to be in a specific location. A future review aiming to include treatments for this age group could focus on computerized program treatments rather than internet-delivered treatments.

This review revealed deficient reporting of comorbid conditions experienced by the participants, which is problematic because of the high prevalence in the patient group [31]. It is reasonable to assume that there were participants with comorbidities or secondary diagnoses in most of the studies as long as these comorbid conditions were not used as an exclusion criterion. Reports on this should be included in future studies. In the studies where this was reported (84/111, 75.7%), DSM-IV Axis I disorders, especially anxiety disorders, were the most prevalent. Given the high prevalence of comorbid anxiety with depressive disorder [32-34], this was as expected. A total of 0.9% (1/111) of the studies [134] excluded participants with symptoms of anxiety disorders or posttraumatic stress disorder, which could limit the ability to generalize the findings. Testing treatments directed at patients without other comorbid DSM-IV Axis I disorders could, by contrast, lead to highly targeted treatments. Specialized and individualized treatment programs could potentially reduce the problem of treatment dropout, that is, assuming that the participant continues to deem all modules or lessons as highly relevant for treatment success. Further research on how comorbid conditions affect the response to treatment and how treatment programs should be amended is needed.

This scoping review led to the identification of several studies (22/111, 19.8%) in which participants had physical diseases or disorders. For patients with chronic pain, diabetes, cancer, or renal disease or kidney failure, the flexibility and accessibility of the internet-delivered format could be crucial for implementation of the treatment.

#### Treatment Completion

A minority of the studies (22/111, 19.8%) provided a clearly stated definition of what was considered an adequate dosage of the treatment for it to be considered completed. It is problematic if treatment programs are not evaluated with the aim of ensuring access to effective treatment components. If it is expected that participants will not interact with all the modules or lessons in the program, it could be argued that the treatments should be made shorter and only include the most effective components. It is also problematic if most studies make conclusions regarding treatment efficacy or effectiveness based on a study sample where dosage or interaction with treatment were not evaluated.

Most studies provided information about the number of participants who completed all modules or lessons in the program. As a result, we synthesized this information although these data could be a somewhat problematic measure of treatment adherence [45,46]. Several studies included optional modules that were directed at specific problems (eg, insomnia) that might not have been applicable to all participants. Treatment adherence is considered a challenge for both psychotherapy in general [41] and digital psychotherapeutic treatments [36-39]. Thus, it would be advantageous to have some consensus when reporting nonadherence to treatment to be able to effectively target the challenging areas of nonadherence. To compare across studies, an operationalization of treatment completion and reporting of the number of participants considered completers are needed in future studies.

A minority of the studies (17/111, 15.3%) provided reasons for noncompletion of the treatment; this is in line with previous findings [39,44]. The most reported reasons were primarily associated with the participants’ personal experiences and everyday challenges. These reasons might be expected from all participants. Other reasons such as reports of technical difficulties and the treatment not meeting personal needs and preferences were provided as rationales for nonadherence to treatment programs. This information could be of high value when developing and refining digital treatments. Dropout rates because of aspects of the treatment should be addressed, and the collection of data from participants who drop out is a necessary future step in this line of research.

In 47% (8/17) of these studies, treatment dropout as a consequence of recovery was reported [87,88,134-139]. It would be highly interesting to assess whether dropout as a result of recovery was also reported in 100% (111/111) of the studies. This lack of reporting is a problem that is discussed in other reviews [44]. If a large number of participants drop out because of recovery early in treatment, it could indicate that some of the components were highly effective or that the participants were misdiagnosed. Furthermore, dropout because of recovery could also indicate that it would be advantageous to deliver the treatment program in a stepped care model where treatment and data collection are conducted with the expectation that participants will recover during the treatment and therefore, should be considered treatment completers. A stepped care model would also make it possible to reduce the resources spent on each patient, and guidance by a therapist could be delivered only to participants who do not recover when the treatment is delivered unguided.

### Limitations

The first author screened the results from the literature search and extracted and charted the data without reliability measures being conducted. The fifth author (ANH) was consulted when the eligibility of a study was unclear, but the studies discussed were selected by the first author. This is a limitation of this scoping review. It is possible that screening, extraction, and charting would have led to different results if conducted by several researchers.

In an effort to make the treatments included in the scoping review comparable, only guided treatments were included. However, it is important to acknowledge that there is variation in the guidance offered in the different studies. In addition to differences in who delivered the guidance, the frequency, modality, and content of the guidance also varied across the studies. In some studies, guidance was available exclusively upon request (eg, the study by Beiwinkel et al [140]) and, in other studies, guidance was initially delivered to all participants and thereafter available only upon request (eg, the studies by Kladnitski et al [75] and Smith et al [141]). Other studies included weekly feedback with brief emails (eg, the study by Ünlü Ince et al [124]), 5- to 10-minute therapist contact (eg, the study by Mullin et al [142]), or phone calls or instant messaging (eg, the study by Dear et al [143]). The large variation in guidance and the therapist’s role in the treatment make the methods used in the different studies less comparable. It is possible that participants in studies with guidance solely upon request completed the entire treatment unguided.

The comprehensiveness of the study was also reduced owing to our restrictions on the year of publication. However, previous years have been thoroughly covered in other reviews. Despite this limitation, we attempted to provide an extensive and large sample of studies in our review. There are also a limited number of studies on internet-delivered treatments in the initial years of the internet, which could also be related to participants’ limited availability and access to the internet in general.

Other limitations of our findings are the exclusion of studies published in languages other than English or one of the Scandinavian languages and the exclusion of gray literature or literature pieces that were not formally published. By excluding gray literature, there is a chance of publication bias in that studies considered to have novel or significant results will have a greater chance of being published [190].

### Future Review Studies

A scoping review where inclusion is not limited to controlled studies could potentially provide a comprehensive overview of the research area. Studies investigating how a treatment works could lead to a lot of additional and important information [47]. Therefore, inclusion of these types of studies in future reviews would be beneficial.

Given our restriction to only include stand-alone internet-delivered treatments in this scoping review, several studies were consequently excluded. Therapies in which internet-delivered treatment is provided in combination with other treatments could lead to efficient and effective outcomes for participants with reduced costs and use of resources. This cost-effectiveness of treatment and reduction in use of resources could allow for an increase in participants’ access to needed services and treatment therapies. For example, patients with more severe symptoms of depression could have greater access to needed therapy in combination with the already existing treatment practices. In future reviews, the inclusion of studies in which the internet-delivered treatment is used as a supplement to other treatments would be beneficial to assess. Comparisons between stand-alone and supplemental treatments could also be an area of interest.

A potential interesting item to investigate could be how the treatments were made available to the participants. As we included all studies independent of how the treatment was accessed by the participants (as long as it was delivered over the internet), all formats were included in this review but not described in detail. We believe that treatment delivery via web-based programs, websites, or apps might not represent vastly different user experiences (eg, the difference between opening an app on a smartphone and visiting a website on the same device could be minimal). At the same time, usability, design, user interface, and functionality of the treatment program could be of great importance, and this could be an interesting topic to investigate for a future study.

Studies examining the preventive effect of internet-delivered treatment for depressive symptoms would also make a highly relevant and interesting area for a future review. If cases of depression could be prevented using low-cost and highly accessible methods of treatment delivered over the internet, this would be both ethically and economically beneficial, and research on this area should be prioritized.

### Conclusions

A total of 111 studies on guided internet-delivered treatment were suitable for inclusion in this scoping review. The RCT design was the most prevalent, and the use of other designs and active control conditions is recommended for future studies. Variations in the outcome measurement scales applied were identified, and more consistency could be beneficial. Lack of accessibility to treatment manuals and few replications where the same treatment program was tested were also discovered and discussed. In future research, underrepresented populations (men, teenagers or young adults, older adults, and non-Western populations) should be included to a greater extent. Given the high prevalence of comorbidities in participants with depressive disorder, the reports of comorbidities in the included studies were somewhat deficient, and future research should investigate this further. Few studies provided definitions of when the treatments were considered completed (22/111, 19.8%) and reasons for treatment dropout (17/111, 15.3%). Most studies (89/111, 80.2%) provided information about the number of participants who completed all treatment modules or lessons or fulfilled a stated definition of treatment completion. There were more studies identified in which >51% of the participants completed the treatment compared with studies in which <50% of the participants completed the treatment. Finally, in only 4.5% (5/111) of the studies, >90% of the participants completed the treatment.

## Appendix

Multimedia Appendix 1 Coding manual.

Multimedia Appendix 2 Search terms and Boolean operators.

Multimedia Appendix 3 Screened reviews.

Multimedia Appendix 4 Data items extracted and charted from the included studies.

Multimedia Appendix 5 All the included studies.

## Abbreviations

CBT: cognitive behavioral therapy
DSM-IV: Diagnostic and Statistical Manual of Mental Disorders, Fourth Edition
PRISMA: Preferred Reporting Items for Systematic Reviews and Meta-Analyses
PRISMA-ScR: Preferred Reporting Items for Systematic Reviews and Meta-Analyses extension for Scoping Reviews
RCT: randomized controlled trial
RQ: research question

## Data Availability

The original contributions presented in this study are included in the paper and appendixes. Further inquiries can be directed to the corresponding author.
