# Supplementary material for: Guided Internet-Delivered Treatment for Depression: Scoping Review
Source: JMIR Ment Health. 2022 Oct 4;9(10):e37342. doi: 10.2196/37342 (PMC9579933; doi:10.2196/37342)
Supplement: Multimedia Appendix 1 [file mental_v9i10e37342_app1.docx]

**Appendix 1**

**Coding manual: criteria for including and excluding studies**

***Study designs***

**Include.** Quantitative studies with a randomized experimental or quasi- experimental design with a control group and single-case design with a control condition (e.g. multiple baseline design with three or more participants).

**Exclude.** Reviews, meta-analysis and non-experimental articles like news stories about studies, commentaries and letters to the editor etc.

Qualitative studies.

***Outcome measures***

**Include.** Studies where at least one of the primary outcome measures is a standardized test for depressive symptoms: e.g. Patient Health Questionnaire-9 (PHQ-9), Geriatric Depression Scale (GDS), the Beck Depression Inventory-II (BDI-II).

In studies where patients with several disorders are included, e.g. some patients with anxiety disorder and others with depression, the outcome measures from the initially depressed patients should be separate from measures from the other patients (if combined PHQ-9 scores from both groups are reported, exclude the study).

**Exclude.** Studies where primary outcome measures are tests measuring symptoms related to other diseases, therapist behavior, well-being, perceived stress or patients characteristics (age, comorbidity, income, marital status etc.)

Studies where the primary outcome is drop-out rates, usability or other properties of the treatment intervention.

Studies with non-standardized tests for reporting depressive symptoms.

Studies measuring cost-effectiveness of interventions (as primary outcome measure) should also be excluded.

***Types of treatments***

**Include.** Treatments delivered digitally: web-page, online program, phone-apps etc, where the participants get access to the treatment from their own home or anywhere with internet connection.

There are no limitations to the psychological approach for the treatment (mindfulness, behavioural activation, CBT, interventions to promote exercise and healthy lifestyle etc. could all be included).

The internet-delivered treatment should be a stand-alone intervention and not a supplement to other treatments.

Guided treatments. At least as an optional possibility, support from another human (therapist, coach or other ***trained*** support staff) should be included in the intervention. The guidance should be implemented in the whole treatment and not limited to measurements and testing (e.g. pre and post-tests with PHQ-9) or reminders to complete online tasks.

**Exclude.**

Studies where the treatments are non-digital, and the participants' access to the intervention requires physical presence at a certain location for the main part of the intervention (e.g. treatments in schools using school computers).

Telehealth-treatments, where main parts of the treatment are delivered through phone calls/ video calls. Treatments where the main part is delivered over the internet, but support is offered by phone calls should be included.

Preventive interventions, interventions to promote well-being and life-style changes, treatments directed to symptoms of comorbid disorders (anxiety, insomnia, chronic pain etc.)

Unguided treatments, where the treatment is fully automated and/or feedback is limited to pre-produced messages.

Treatments where guidance and support are offered from untrained individuals (e.g. online support groups where participants support each other) should be excluded.

Studies directed at the behavior of the therapists or others who implement the treatment.

***Types of participants***

**Include.**

Studies with participants who initially have at least a mild depressive disorder (measured on a standardized test), with or without comorbid disorders.

Studies directed at women suffering from maternal depression.

No limitations regarding participants' age, gender or living conditions.

**Exclude.**

Studies with participants with subthreshold depression. Studies with participants defined as at risk for depression, not currently depressed: e.g. caregivers or parents in difficult care situations, people experiencing medical or other trauma (loss of child, cancer, COVID-19, natural disasters, terror etc.) and other groups where interventions are implemented to promote psychological well-being (students, military personnel etc.).

Studies with participants with bipolar disorder should also be excluded.
