# Supplementary material for: Guided Internet-Delivered Treatment for Depression: Scoping Review
Source: JMIR Ment Health. 2022 Oct 4;9(10):e37342. doi: 10.2196/37342 (PMC9579933; doi:10.2196/37342)
Supplement: Multimedia Appendix 2 [file mental_v9i10e37342_app2.docx]

**Appendix 2**

**Search terms and boolean operators**

The search in the databases (PubMed, Scopus, PsycINFO, MEDLINE and ERIC) were limited to empirical studies in peer reviewed journals published between January 1, 2010 and January 13, 2021.

The following search terms and boolean operators were used:

(Online treatment **OR** Internet delivered treatment **OR** Internet-based treatment **OR** Computerized treatment **OR** Digital treatment **OR** VR treatment **OR** Virtual reality treatment **OR** Online intervention **OR** Internet delivered intervention **OR** Internet-based intervention **OR** Computerized intervention **OR** Digital intervention **OR** VR intervention **OR** Virtual reality intervention **OR** Online therapy **OR** Internet delivered therapy **OR** Internet-based therapy **OR** Computerized therapy **OR** Digital therapy **OR** VR therapy **OR** Virtual reality therapy **OR** Online program **OR** Internet delivered program **OR** Internet-based program **OR** Computerized program **OR** Digital program **OR** VR program **OR** Virtual reality program **OR** Online self-help **OR** Internet delivered self-help **OR** Internet-based self-help **OR** Computerized self-help **OR** Digital self-help **OR** VR self-help **OR** Virtual reality self-help **OR** E-health **OR** iCBT [internet cognitive behavioural therapy] **OR** Internet CBT)

**AND**

(Guided **OR** Therapist guided **OR** Feedback **OR** Coach* **OR** Monitor* **OR** Support*)

**AND**

**(**Depress* **OR** Depressive*).
