# Supplementary material for: Guided Internet-Delivered Treatment for Depression: Scoping Review
Source: JMIR Ment Health. 2022 Oct 4;9(10):e37342. doi: 10.2196/37342 (PMC9579933; doi:10.2196/37342)
Supplement: Multimedia Appendix 3 [file mental_v9i10e37342_app3.docx]

**Appendix 3**

**Reviews screened for relevant studies to be included in the scoping review**

Andersson, G., Cuijpers, P., Carlbring, P., Riper, H., & Hedman, E. (2014). Guided Internet‐based vs. face‐to‐face cognitive behavior therapy for psychiatric and somatic disorders: a systematic review and meta‐analysis. *World Psychiatry, 13*(3), 288-295. https://doi.org/10.1002/wps.20151

Arnberg, F. K., Linton, S. J., Hultcrantz, M., Heintz, E., & Jonsson, U. (2014). Internet-delivered psychological treatments for mood and anxiety disorders: a systematic review of their efficacy, safety, and cost-effectiveness. *PLOS ONE, 9*(5), e98118. https://doi.org/10.1371/journal.pone.0098118

Baumeister, H., Reichler, L., Munzinger, M., & Lin, J. (2014). The impact of guidance on Internet-based mental health interventions: A systematic review. *Internet Interventions, 1*(4), 205-215. https://doi.org/10.1016/j.invent.2014.08.003

Carlbring, P., Andersson, G., Cuijpers, P., Riper, H., & Hedman-Lagerlöf, E. (2018). Internet-based vs. face-to-face cognitive behavior therapy for psychiatric and somatic disorders: an updated systematic review and meta-analysis. *Cognitive Behaviour Therapy, 47*(1), 1-18. https://doi.org/10.1080/16506073.2017.1401115

Davies, E. B., Morriss, R., & Glazebrook, C. (2014). Computer-delivered and web-based interventions to improve depression, anxiety, and psychological well-being of university students: a systematic review and meta-analysis. *Journal of Medical Internet Research, 16*(5), e130. https://doi.org/10.2196/jmir.3142

Dowling, M., & Rickwood, D. (2013). Online counseling and therapy for mental health problems: A systematic review of individual synchronous interventions using chat. *Journal of Technology in Human Services, 31*(1), 1-21. https://doi.org/10.1080/15228835.2012.728508

Etzelmueller, A., Vis, C., Karyotaki, E., Baumeister, H., Titov, N., Berking, M., Cuijpers, P., Riper, H., & Ebert, D. D. (2020). Effects of internet-based cognitive behavioral therapy in routine care for adults in treatment for depression and anxiety: Systematic review and meta-analysis. *Journal of Medical Internet Research, 22*(8), e18100. https://doi.org/10.2196/18100

Fleming, T. M., Cheek, C., Merry, S. N., Thabrew, H., Bridgman, H., Stasiak, K., Shepherd, M., Perry, Y., & Hetrick, S. (2014). Serious games for the treatment or prevention of depression: a systematic review. *Revista de Psicopatología y Psicología Clínica, 19*(3), 227–242. https://doi.org/10.5944/rppc.vol.19.num.3.2014.13904

Garrido, S., Millington, C., Cheers, D., Boydell, K., Schubert, E., Meade, T., & Nguyen, Q. V. (2019). What works and what doesn’t work? A systematic review of digital mental health interventions for depression and anxiety in young people. *Frontiers in Psychiatry, 10*, 759. https://doi.org/10.3389/fpsyt.2019.00759

Hedman, E., Ljótsson, B., & Lindefors, N. (2012). Cognitive behavior therapy via the Internet: a systematic review of applications, clinical efficacy and cost–effectiveness. *Expert review of pharmacoeconomics & outcomes research, 12*(6), 745-764. https://doi.org/10.1586/erp.12.67

Huguet, A., Rao, S., McGrath, P. J., Wozney, L., Wheaton, M., Conrod, J., & Rozario, S. (2016). A systematic review of cognitive behavioral therapy and behavioral activation apps for depression. *PLOS ONE, 11*(5), e0154248. https://doi.org/10.1371/journal.pone.0154248

Karyotaki, E., Ebert, D. D., Donkin, L., Riper, H., Twisk, J., Burger, S., Rozental, A., Lange, A., Williams, A. D., & Zarski, A. C. (2018). Do guided internet-based interventions result in clinically relevant changes for patients with depression? An individual participant data meta-analysis. *Clinical Psychology Review, 63*, 80-92. https://doi.org/10.1016/j.cpr.2018.06.007

Königbauer, J., Letsch, J., Doebler, P., Ebert, D., & Baumeister, H. (2017). Internet- and mobile-based depression interventions for people with diagnosed depression: A systematic review and meta-analysis. *Journal of Affective Disorders, 223,* 28-40. https://doi.org/10.1016/j.jad.2017.07.021

Lee, E. W., Denison, F. C., Hor, K., & Reynolds, R. M. (2016). Web-based interventions for prevention and treatment of perinatal mood disorders: a systematic review. *BMC pregnancy and childbirth, 16(*1), 1-8. https://doi.org/10.1186/s12884-016-0831-1

Li, J., Theng, Y.-L., & Foo, S. (2014). Game-based digital interventions for depression therapy: a systematic review and meta-analysis*. Cyberpsychology, Behavior, and Social Networking, 17*(8), 519-527. https://doi.org/10.1089/cyber.2013.0481

Loughnan, S. A., Joubert, A. E., Grierson, A., Andrews, G., & Newby, J. M. (2019). Internet-Delivered psychological interventions for clinical anxiety and depression in perinatal women: a systematic review and meta-analysis*. Archives of Women's Mental Health, 22*(6), 737-750. https://doi.org/10.1007/s00737-019-00961-9

Martínez, P., Rojas, G., Martínez, V., Lara, M. A., & Pérez, J. C. (2018). Internet-based interventions for the prevention and treatment of depression in people living in developing countries: a systematic review. *Journal of affective disorders, 234*, 193-200. https://doi.org/10.1016/j.jad.2018.02.079

Paganini, S., Teigelkoetter, W., Buntrock, C., & Baumeister, H. (2018). Economic evaluations of internet-and mobile-based interventions for the treatment and prevention of depression: a systematic review. *Journal of affective disorders, 225*, 733-755. https://doi.org/10.1016/j.jad.2017.07.018

Păsărelu, C. R., Andersson, G., Bergman Nordgren, L., & Dobrean, A. (2017). Internet-delivered transdiagnostic and tailored cognitive behavioral therapy for anxiety and depression: a systematic review and meta-analysis of randomized controlled trials. *Cognitive Behaviour Therapy, 46*(1), 1-28. https://doi.org/10.1080/16506073.2016.1231219

Pennant, M. E., Loucas, C. E., Whittington, C., Creswell, C., Fonagy, P., Fuggle, P., Kelvin, R., Naqvi, S., Stockton, S., & Kendall, T. (2015). Computerised therapies for anxiety and depression in children and young people: a systematic review and meta-analysis. *Behaviour research and therapy, 67*, 1-18. https://doi.org/10.1016/j.brat.2015.01.009

Rice, S. M., Goodall, J., Hetrick, S. E., Parker, A. G., Gilbertson, T., Amminger, G. P., Davey, C. G., McGorry, P. D., Gleeson, J., & Alvarez-Jimenez, M. (2014). Online and social networking interventions for the treatment of depression in young people: a systematic review. *Journal of Medical Internet Research, 16*(9), e206. https://doi.org/10.2196/jmir.3304

Richards, D., & Richardson, T. (2012). Computer-based psychological treatments for depression: a systematic review and meta-analysis. *Clinical Psychology Review, 32*(4), 329-342. https://doi.org/10.1016/j.cpr.2012.02.004

Rosenbaum, S., Newby, J. M., Steel, Z., Andrews, G., & Ward, P. B. (2015). Online physical activity interventions for mental disorders: A systematic review. *Internet Interventions, 2*(2), 214-220. https://doi.org/10.1016/j.invent.2015.04.001

Saddichha, S., Al-Desouki, M., Lamia, A., Linden, I. A., & Krausz, M. (2014). Online interventions for depression and anxiety–a systematic review. *Health Psychology and Behavioral Medicine: An Open Access Journal, 2*(1), 841-881. https://doi.org/10.1080/21642850.2014.945934

Sztein, D. M., Koransky, C. E., Fegan, L., & Himelhoch, S. (2018). Efficacy of cognitive behavioural therapy delivered over the Internet for depressive symptoms: A systematic review and meta-analysis. *Journal of telemedicine and telecare, 24*(8), 527-539. https://doi.org/10.1177/1357633x17717402

Tokgöz, P., Hrynyschyn, R., Hafner, J., Schönfeld, S., & Dockweiler, C. (2021). Digital Health Interventions in Prevention, Relapse, and Therapy of Mild and Moderate Depression: Scoping Review. *JMIR Mental Health, 8*(4), e26268. https://doi.org/10.2196/26268

Xiang, X., Wu, S., Zuverink, A., Tomasino, K. N., An, R., & Himle, J. A. (2020). Internet-delivered cognitive behavioral therapies for late-life depressive symptoms: a systematic review and meta-analysis. *Aging & Mental Health, 24*(8), 1196-1206. https://doi.org/10.1080/13607863.2019.1590309

Ye, X., Bapuji, S. B., Winters, S. E., Struthers, A., Raynard, M., Metge, C., Kreindler, S. A., Charette, C. J., Lemaire, J. A., & Synyshyn, M. (2014). Effectiveness of internet-based interventions for children, youth, and young adults with anxiety and/or depression: a systematic review and meta-analysis. *BMC health services research, 14*(1), 1-9. https://doi.org/10.1186/1472-6963-14-313
