# Supplementary material for: Guided Internet-Delivered Treatment for Depression: Scoping Review
Source: JMIR Ment Health. 2022 Oct 4;9(10):e37342. doi: 10.2196/37342 (PMC9579933; doi:10.2196/37342)
Supplement: Multimedia Appendix 4 [file mental_v9i10e37342_app4.docx]

**Appendix 4**

**Data items extracted and charted from the included studies**

***Items related to RQ 1. How are the studies designed?***

**Design.** Research designs were charted based on statements in the articles.

**Control condition.** Based on the definitions provided by Cochrane Handbook for Systematic Reviews of Interventions [71] and Lindquist et al [72] we evaluated and charted whether the control conditions used in the studies were active or inactive/passive. No treatment, wait-list control, attention control and treatment as usual was classified as (1) “Inactive control”. Access to other treatments, or variations (e.g., internet-delivered treatment without guidance) or parts of the treatment (e.g., access to textual material, but not feedback or tasks) were classified as (2) “Active control”. (3) ”Active and inactive control” were used to classify studies with more than one control condition, where at least one was an active control and at least one was an inactive control.

**Efficacy/ effectiveness.** We charted whether the studies were described by the authors as efficacy or effectiveness studies. Studies without statements about efficacy or effectiveness were charted as “not defined”. We made no interpretations and charted solely based on statements in the research papers.

**Outcome measure.** The outcome measures were charted based on descriptions of primary or main outcome measures. For studies where several measures for depressive symptoms were applied and defined as primary outcome measures all were chartered. Use of other measures assessing other symptoms not related to depression (e.g., Generalized Anxiety Disorder scale [GAD-7] etc.) were not charted even though they were defined as one of the primary outcome measures.

***Items related to RQ 2. What type of treatments are delivered?***

**Treatment approach.** Based on the descriptions of the treatments, we extracted the approach or techniques used in the *relevant conditions*. A relevant condition was a condition where participants received guided internet-delivered treatment, the guide was trained, and measurements of depressive symptoms was conducted. Some study arms did not fulfill these criterias (e.g. if the treatment offered in comparison groups was delivered face-to-face or study arms where the participants had a diagnosis other than depression), and data was not extracted for these study arms.

The definition of approaches was charted solely based on how it was defined by the authors of the studies. All approaches were charted for studies where a combination of approaches was used. For studies where treatments based on different approaches were delivered in more than one relevant study condition we charted each approach separately.

**Treatment manual.** We searched the method section and appendix/ supplementary material to the studies for access to treatment manuals, and charted if they were easily accessible without extensive backtracking.

**Name of treatment.** If provided, we charted the name of the treatment program used to compare if the same treatments were used in several studies.

**Guidance.** The guidance offered in the studies were sorted into three categories based on the guides professional background and education; (1) trained professionals (psychiatrist, psychologist, nurses, mental health workers, social workers, counselors and other support personnel that had received some courses or training), (2) research staff (authors, research assistants), and (3) students (bachelor, masters and ph.d level, psychotherapists-in-training).

For studies with several guides, where the guides had varying backgrounds the studies were categorized into the first of the categories (1), (2) and (3) that were applicable for one of the guides.

**Duration.** The number of weeks needed to complete the treatments were charted. For studies where duration was described in a given number of completed lessons, sessions or modules this was charted. For studies where each lesson/session or module were estimated to take a given amount of time (e.g. one lesson per week), the time needed to complete the whole treatment was calculated.

***Items related to RQ 3. What are the characteristics of the participants in the studies?***

**Number of participants.** The number of participants in relevant study conditions were charted. In studies with several relevant conditions, the number of participants were added together. Participants in non-relevant conditions were not charted.

**Gender of participants.** If reported, we extracted data on gender representation in the relevant conditions. The number/percentage of female participants were charted (with a simplification made that all participants that were not reported to be female were male).

**Age of participants.** Mean age in the relevant study conditions were charted. For studies with more than one relevant condition, the mean age in each relevant condition was added together and divided by the number of relevant conditions in that study.

**Age-group in focus.** We charted information on the age-group in focus in four categories based on statements by the authors or interpretations based on the criteria for inclusion described in the studies.

(1) Children: studies that included children up to 12 years of age, described the age group in focus to be children, or where it was reasonable to assume that the participants were between 0 and 12 years of age.

(2) Teenagers and young adults: studies that included participants described as young adults, adolescents, teenagers or participants between 12 and 30 years of age. This group also included participants that due to their affiliation to the population in focus were assumed to be between 12 and 30 years of age (e.g. university students).

(3) Adults: studies that included participants described as adults, or participants over 17 years of age. If there was an upper age limit for participation this had to be higher than 30 years, to separate this group from category 2. This group also included participants that due to their affiliation to the population in focus were assumed to be over 17 years (e.g. caregivers of adolescents, army veterans).

(4) Older adults: Studies that excluded participants under 60 years of age, described the age-group in focus to be older adults, or where it was reasonable to assume that the participants were over 60 years of age.

**Comorbid disorders.** All information about DSM-IV Axis I disorders and somatic disorders experienced by the participants were charted. Where comorbidity was not reported, we used the labels “not reported” or “included, but not described in detail”. The label “anxiety” was used for all anxiety disorders (as defined by DSM-5[7]).
 **Countries of recruitment.** We extracted the countries participants were recruited from. If the country was not specifically stated in the description of the recruitment process, assumptions were made based on the authors affiliation, description of founding or ethical approvals, and/or language used in the treatment.

***Items related to RQ 4. What is the treatment completion in the various studies?***

**Definitions of treatment completion.** We charted if treatment completion (adherence to treatment) were defined or not defined.

**Number of completers.** When treatment completion was defined, we collected data on the percentage of participants in relevant conditions that were defined as treatment completers. For studies where this was not defined, we charted the percentage of participants that started and completed the whole treatment (all weeks/modules/lessons).

**Reasons for non-completion.** Data on reasons given for non-completion of the treatment were charted as “given”, “not given”, or “unclear”. For studies where the participants reasons for treatment drop-out where reported, we sorted the reasons provided into eight categories: (1) no reason provided, (2) not meeting personal needs, (3) recovery, (4) preferred other treatment/part of the treatment, (5) technical difficulties, (6) lack of time (7) motivation and (8) personal problems/sickness.

**References**

7. Diagnostic and Statistical Manual of Mental Disorders, 5th edition. Washington, DC: American Psychiatric Publishing; 2013.

71. Cochrane Handbook for Systematic Reviews of Interventions. Hoboken, New Jersey, United States: John Wiley & Sons; 2019.

72. Lindquist R, Wyman J, Talley K, Findorff M, Gross C. Design of control-group conditions in clinical trials of behavioral interventions. J Nurs Scholarsh 2007;39(3):214-221 [FREE Full text] [doi: 10.1111/j.1547-5069.2007.00171.x] [Medline:17760793]
